# Prevalence of congenital colour vision deficiency among Black school children in Durban, South Africa

**DOI:** 10.1186/s13104-019-4374-1

**Published:** 2019-06-10

**Authors:** Khathutshelo Percy Mashige, Diane Beverly van Staden

**Affiliations:** Discipline of Optometry, School of Health Sciences, University of KwaZulu-Natal, Private Bag X54001, Durban, 4000 South Africa

**Keywords:** Colour vision deficiency, Red-green defects, Prevalence, School children, South Africa

## Abstract

**Objectives:**

Congenital colour vision deficiency (CCVD) is an x-linked chromosome disorder that results from abnormalities in one or all three-cone type’s photoreceptors. Early assessment and diagnosis of CCVD is necessary to minimise the disability associated with the condition. Multistage sampling was used to determine the prevalence of CCVD among Black South African school children in Durban, South Africa. The examination included visual acuity measurements, ocular motility evaluation, retinoscopy, auto-refraction, and examination of the anterior segment, media and fundus. Colour vision testing was performed using Colour Vision Testing Made Easy colour plates (Home Vision Care, Gulf Breeze, FL).

**Results:**

1305 (704 boys and 601 girls) Black school children participated in the study. The overall prevalence of colour vision deficiency was 29 (2.2%), which was higher in boys (25, 4.2%) than girls (4, 0.6%), with prevalence of protanopia and deuteranopia found to be 10 (0.7%) and 19 (1.5%), respectively. The prevalence of protanopia and deuteranopia among males was nine (1.5%) and 16 (2.7%) respectively, which was significantly higher than the 1 (0.1%) protanopia and 3 (0.4%) deuteranopia in females (p < 0.05). Policies and guidelines for comprehensive school eye health programmes which screen children for CCVD are required in South Africa.

**Electronic supplementary material:**

The online version of this article (10.1186/s13104-019-4374-1) contains supplementary material, which is available to authorized users.

## Introduction

Colour vision deficiency is a common disorder of vision that results in the inability to see certain colours or perceive their differences [1]. The prevalence of colour vision deficiency is expected to increase globally with the growing population, and varies among different races and geographical areas [2–4]. It has been reported that most people with colour vision defects remain undiagnosed [3, 4]. Colour vision deficiency is therefore under-reported due to a lack of its awareness and the absence of proper screening to detect this in the general population [4, 5]. Colour vision deficiency can be classified as congenital or acquired, with the former being a non-progressive, untreatable disorder that is constant throughout life [6, 7]. Acquired colour vision defects are secondary to ocular or general pathology, such as neurological disease, some metabolic disorders, drug toxicity and prolonged exposure to certain solvents and medications [5].

Persons with normal colour vision have three types of retinal cone photoreceptors that aid in perceiving red, green and blue colours [4]. Those with abnormal cones will perceive colours differently [4]. There are various types of colour vision deficiency namely monochromacy, dichromacy and trichromacy [4]. Monochromacy is complete colour blindness, this rare condition occurring when two or three of the cone pigments are missing [4, 8]. Dichromacy occurs when a person only has two retinal cones types that are able to perceive colour, resulting in the total absence of one colour. “*Dichromacy includes protanopia (caused by the complete absence of red retinal photoreceptors), deuteranopia (caused by the absence of green retinal photoreceptors) and tritanopia (complete absence of blue retinal receptors*) [8]”. “*In anomalous trichromacy, one of the three cone pigments is altered in its spectral sensitivity and includes protanomaly (involves reduced sensitivity to red), deuteranomaly (involves reduced sensitivity to green) and tritanomaly in which the spectral sensitivity of the red, green and blue/yellow receptors is altered* [8]”.

Although statistics vary across different groups and populations, the prevalence of CCVD in European Caucasians is approximately 8% in men and 0.4% in women, with between 4 and 6.5% prevalence in men of Chinese and Japanese ethnicity [9]. The prevalence of CCVD has also been reported to be rising in men of African ethnicity [9]. Although CCVD is incurable and does not cause complete blindness [10], those with the defect (except a few mildly affected deuteranomals) have reported problems in everyday life and at work [5, 11]. For instance, occupations where CCVD is a disadvantage include art teaching, interior design, histopathology, horticulture, geology, diamond grading and metallurgy [12]. Persons with CCVD should, therefore, ideally know about their condition so that they can be advised on appropriate career choices, especially during their early education years. However, Cole [13] reported that many school children are unaware of their colour vision status, which could negatively impact on their school performance. Moreover, early diagnosis of CCVD in children is vital for teachers to make necessary adjustments to the teaching methods for effective and appropriate learning [14]. There is a paucity of published research in South Africa about the prevalence of CCVD among school children. The aim of this study was to determine the prevalence of CCVD Black school children in Durban, South Africa.

## Main text

### Methods and materials

#### Study area and sampling

The participants were recruited from geographically contagious areas of Durban consisting of urban, peri-urban and rural areas. These areas included the South, Inner West Region, and Outer West Regions of the Durban metropolitan area (Additional file 1).

Two field workers assisted in recruiting study participants, with those who agreed to participate in the study being transported by one field worker to the examination site (University of KwaZulu-Natal Optometry Clinic), while the other fieldworker moved to the next selected site. During the field work, the head of the household was informed of the nature and details of the study, the date of the clinical examination as well as transportation arrangements for their children.

### Sampling procedure and sample size

The study participants were selected from the three regions of the Durban area through multistage sampling technique. Simple random sampling was then used to select eight areas from the 14 urban, and eight from the 15 peri-urban/rural areas. Finally, systematic random sampling was used to select study participants. The minimum sample size for the study was calculated by using the formula for estimating a single population proportion [15]:

$${\text{N }} = {\text{ Z}}^{ 2} \times \, \left( {\text{P}} \right) \, \times \, {{\left( { 1- {\text{P}}} \right)} / {{\text{C}}^{ 2} }}$$

where N = minimum required sample size, Z = value of z statistic at 95% confidence level = 1.96, P = assumed prevalence of congenital colour vision defects = 8% for maximum sample size, C = maximum acceptable sampling error = 1.6%.

$${{\left( { 1. 9 6} \right)^{ 2} \times \, \left( {0.0 8} \right) \, \times \, \left( { 1- 0.0 8} \right)} / {\left( {0.0 1 6} \right)^{ 2} }} = { 11}0 5$$

An additional 200 participants were added to compensate for non-response, missing data, sub-group analysis, and to enhance generalisation of findings through a larger sample size, which gave a total sample number of 1305.

#### Data collection procedures

Their socio-demographic details were collected by the field workers through face-to-face interviews at their homes using structured questionnaires with the children and/or their parents/legal guardians. The ocular examination at the clinic consisted of visual acuity measurements, auto-refraction and subjective refraction, pen torch examination, direct ophthalmoscope observation and colour vision testing. The inclusion criterion for this study was school children (aged 7–17) who had a written consent from the parents or guardians. Children with any evidence of ocular pathology, trauma, previous ocular surgery, long-term use of medication and those born to parents who were born outside South Africa were excluded from the study. The pseudo-isochromatic colour plate test, Colour Vision Testing Made Easy (CVTME), was used to assess the colour vision of all the participants. All the testing was conducted under binocular viewing conditions in an optometry clinic with standard illumination, the details of the test process having been described previously [16].

#### Data analysis

Data were entered into excel spreadsheet and then exported to the Statistical Package for Social Sciences (SPSS version 24) for analysis. Descriptive statistics was used to present the participant’s socio-demographic characteristics. Chi square (χ^2^) test was used to establish the statistical significances, with p-values of less than 0.05 being considered statistically significant.

### Results

A total of 1305 school children participated in the study, their ages ranging from 7–17 years, with a mean (± standard deviation) of 12.06 (± 1.8) years. There were 704 (53.9%) females and 601 (46.1%) males, while 719 children (55.1%) were aged 7–12 years and 586 (44.9%) were aged 13–17. The findings of CCVD among the 1305 subjects are shown in Table 1. The prevalence of CCVD was 29 (2.2%) (95% CI 0.9–3.1), which was higher in males (25, 4.2%) (95% CI 3.1–6.4) than females (4, 0.6%) (95% CI 0.1–1.1). There was a statistically significant difference in the prevalence of CCVD for boys (p = 0.00), but not for girls (p = 0.14). There were more (19, 1.5%) deutans than protans (10, 0.7%). Similarly, there were significantly more protans (9, 1.5%) and deutans (16, 2.7%) in boys than girls, who had one (0.1) protan and three (0.4%) deutans (p < 0.05). No child was totally colour blind among the study population. The prevalence of CCVD was compared among younger and older children, although the difference between the age groups was not statistically significant (p = 0.12).

**Table 1:** Prevalence of CCVD according to age and gender

Category	Total n (%)	Protans n (%)	Deutans n (%)	CCVD n (%) [95% CI]	p-value
Age (years)					
7–12	719	7 (1.0)	6 (0.8)	13 (1.8) [0.7–2.8]	0.12
13–17	586	3 (0.5)	13 (2.2)	16 (2.7) [1.1–4.2]	
Gender					
Male	601 (46.1)	9 (1.5)	16 (2.7)	25 (4.2) [3.1–6.4]	0.00
Female	704 (53.9)	1 (0.1)	3 (0.4)	4 (0.5) [0.1–1.1]	
Total	1305	10 (0.7)	19 (1.5)	29 (2.2) [0.9–3.1]	

### Discussion

Irrespective of age, race and ethnicity, there is a general paucity of information on the colour vision status of South Africans. Colour vision assessments are important to enable those affected to follow adaptive strategies that could minimise the risks associated with the disorder. This, the first report on CCVD in healthy Black South Africans school children, provides a basic database on the prevalence of CCVD in the region and an opportunity to compare data with other race and ethnic groups from a number of countries.

Congenital red-green colour vision defects are the most common types of colour vision deficiencies [16]. Colour Vision Testing Made Easy (CVTME) is the paediatric gold standard for identifying and classifying red-green colour vision defects in children as young as 3 years [16, 17], with a mean sensitivity of 91%, a mean specificity of 100%, in addition to having good retest reliability [17]. The distribution of CCVD was consistent across the age categories (7–12: 1.8%, 13–17: 2.7%), and although this shows an increase in the prevalence of the defect with increasing age, the difference was not statistically significant (p = 0.12). As CCVD is a hereditary defect, the prevalence in different age groups is statistically insignificant (p > 0.05). Table 2 provides an overview of the prevalence of CCVD in selected studies among individuals of different ages, races and ethnicities compared with findings from this study.

**Table 2:** Characteristics of congenital colour vision defects (CCVD) reported compared with the findings of the current study

Study	Race	Instrument used	Number	Age	Overall prevalence (%)	Prevalence (95% CI)	
			M/F			M	F
Africa							
This study	Zulu	CVTME	704/601	7–17	2.2	4.2	0.6
Ugalahi et al. [18]	Nigerian	Ishihara, FM D-15	769/866	13.9 ± 1.9	2.3	3.8	0.9
Tabansi et al. [19]	Nigerian	Ishihara	N/R	N/R	2.6	N/R	N/R
Abah et al. [21]	Nigerian	Ishihara	149/178	5–17	1.5	N/R	N/R
Woldeamanuel and Geta [23]	Ethiopian	Ishihara	844	7–18 (11.75 ± 2.5)	4.1	3.6	0.6
Mulusew and Yilikal [24]	Ethiopian	Ishihara	1040	12 ± 2.43	4.2 (95% CI 2.98–5.42)	N/R	N/R
Asia/Middle East							
Ahsana et al. [5]	Iranian	Ishihara	1352/1302	N/R	N/R	8.73	1.69
Reddy et al. [22]	Indian	Ishihara	841/788	10–15	1.9	1.71	0.814
Rajavi et al. [26]	Iranian	Ishihara	1069/1081	7–12 (mean, 9.4 ± 1.7)	2.2% (95% CI 1.5–3)	3.5	1
Niroula and Saha [2]	Chhetri	Ishihara	474/490	10–19	N/R	3.8	0
Shrestha et al. [20]	Chhetri	Ishihara	1050/951	10.35 ± 2.75 M 10.54 ± 2.72 F	2.1	3.9	0
Napaporn et al. [27]	Thai	Ishihara	3056	6–7	4.2	3.7	0.5
Europe							
Gallo et al. [28]	Italian	Ishihara	63 933	11–14	4.8		
Azizoğlu et al. [29]	Turkish	Ishihara	823	4–10	3.8		
Rose et al. [30]	Caucasian	Ishihara	131	5–18	5.3		
Birch and Platts [31]	Caucasian	Ishihara	258/255	3–11	N/R	6.6	0.4

*M* male, *F* female, *CI* confidence interval, *N/R* not reported

Total congenital colour vision deficiency was not detected in this study, which confirms the view that the trait, which is autosomal, is rare [1]. The prevalence of CCVD in this study was 2.2%, which is similar to the prevalence rates of 2.3% in Ibadan, South-West Nigeria [18], 2.6% in Port-Harcourt, Southern Nigeria [19], 1.9% [2] and 2.1% [20] in Nepal, and slightly higher than 1.5% found in Zaria, Northern Nigeria [21] among school children. This finding is also comparable with that of Guntur City, Andhra Pradesh, which reported a prevalence rate of 1.9% [22]. However, the result of this study is lower than the prevalence rate of 4.1% and 4.2% reported in Southern [23] and Central Ethiopia [24] respectively among school children. This finding is also lower than a study done in Manipur, India, which reported a prevalence rate of 5.8% [5]. Differences in study population, geographical area, race, ethnicity and techniques used to assess colour vision could be some of the factors responsible for such variations (Table 2). Higher prevalence rates of CCVD have been reported among Asians and Caucasians compared to African populations [9]. For example, the prevalence of CCVD in Japan was 4%, 6.5% in China, 7.3% in Turkey, 4.7% in Iran and 2.8% to 8.2% in India [9].

The prevalence of CCVD was noted to be higher in males (4.2%) than females (0.5%), which is consistent with other studies [25, 26]. This may be attributed to the fact that CCVD is usually inherited by an X-linked recessive pattern [9]. Therefore, the prevalence rate of CCVD is found to be higher in males as they have only one X-chromosome, and are prone to suffer from the defect while females are carriers [9]. Deutans occurred at a higher frequency than protans among both male and female red-green colour deficient children (Table 1). Similar frequency trends have been reported in earlier studies among different populations [24, 25].

### Conclusion

The prevalence of CCVD among Black school children in Durban was 2.2%, which is comparable to those previously reported in Southwest and South Nigeria, but lower than those in Central and South Ethiopia. These results strengthen the need for local studies to establish complete databases in the African continent. The level of awareness of CCVD can be enhanced by conducting routine colour vision screenings at schools, which will help the affected individuals to recognise their limitation and orientate their choice of future careers.

## Limitations

This study screened only for red-green CCVD as the CVTME is not able to detect blue-yellow deficiency. Children who were diagnosed with CCVD were not retested using another colour vision test with more sensitivity to validate the results. It is recommended that further studies be done to determine the magnitude and severity of CCVD using an anomaloscope. The results of the study are based on 1305 Black school children of isiZulu ethnicity from Durban and might not provide for a complete analysis of Black children across South Africa. It is recommended that other children of South African race and ethnic groups be studied so that comparative inferences can be made.

## Additional file

**Additional file 1.** Map showing the South, Inner West and Outer West regions of Durban. (https://www.google.com/search?q=South,+Inner+West+and+Outer+West+regions+of+Durban&safe=strict&rlz=1C1CHBD_enZA821ZA821&source=lnms&tbm=isch&sa=X&ved=0ahUKEwjpqIXQpcTiAhXWSxUIHbRBDz8Q_AUIECgD&biw=1280&bih=610).

## Data Availability

Not applicable. All the data is in the result.

## Abbreviations

CCVD: congenital colour vision deficiency
CVTME: Colour Vision Testing Made Easy
