# Supplementary material for: Prevalence of congenital colour vision deficiency among Black school children in Durban, South Africa
Source: BMC Res Notes. 2019 Jun 10;12:324. doi: 10.1186/s13104-019-4374-1 (PMC6558866; doi:10.1186/s13104-019-4374-1)
Supplement: Supplementary file 1 — Additional file 1. Map showing the South, Inner West and Outer West regions of Durban. (https://www.google.com/search?q=South,+Inner+West+and+Outer+West+regions+of+Durban&safe=strict&rlz=1C1CHBD_enZA821ZA821&source=lnms&tbm=isch&sa=X&ved=0ahUKEwjpqIXQpcTiAhXWSxUIHbRBDz8Q_AUIECgD&biw=1280&bih=610). [file 13104_2019_4374_MOESM1_ESM.docx]

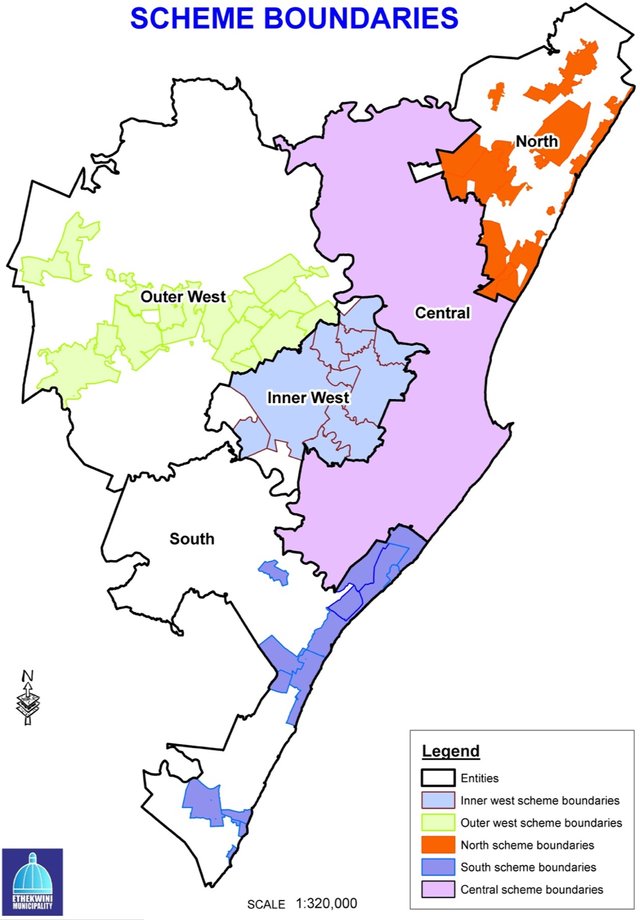


Map showing the South, Inner West and Outer West regions of Durban. (<https://www.google.com/search?q=South,+Inner+West+and+Outer+West+regions+of+Durban&safe=strict&rlz=1C1CHBD_enZA821ZA821&source=lnms&tbm=isch&sa=X&ved=0ahUKEwjpqIXQpcTiAhXWSxUIHbRBDz8Q_AUIECgD&biw=1280&bih=610>)
